# Trapping the Pasture Odorscape Using Open-Air Solid-Phase Micro Extraction, a Tool to Assess Grassland Value

**DOI:** 10.1371/journal.pone.0140600

**Published:** 2015-11-04

**Authors:** Agnès Cornu, Anne Farruggia, Ene Leppik, Centina Pinier, Florence Fournier, David Genoud, Brigitte Frérot

**Affiliations:** 1 UMR1213 Herbivores, INRA—VetAgro Sup—Clermont Université, Saint-Genès Champanelle, France; 2 UMR1392 Institut d’Ecologie et des Sciences de l’Environnement de Paris, INRA, Versailles, France; 3 UE1296 Unité Expérimentale des Monts d’Auvergne, INRA, Laqueuille, France; 4 Diagnostic Gestion Environnement, Arzens, France; 5 Observatoire des Abeilles, Arzens, France; Tsinghua University, CHINA

## Abstract

Besides supporting cattle feeding, grasslands are home to a diversity of plants and insects that interact with each other by emitting volatile compounds. The aim of this work was to develop a method to determine permanent grassland odorscape and relate it to flower-visiting insects. Two grasslands were chosen for their contrasting levels of botanical diversity, resulting from differing grazing managements. Measurements were made over two periods of three consecutive days at the beginning of grazing, and just after the cows had left the plots. Volatile compounds were trapped using solid-phase microextraction (SPME) fibers exposed eight hours a day in three exclosures per plot, and then analyzed by gas-chromatography-mass spectrometry (GC-MS). Insects were trapped using pan traps and a net, sorted and counted. The open air SPME method yielded volatile compound profiles that were richer than maize field profiles, comprising the common green leaf volatiles (GLV) and more specific ones. Differences between the odorscapes of the two grasslands were found, but they were not as marked as expected from their botanical composition. By contrast, there were sharp differences between the two periods, resulting from the combined effects of changes in weather conditions, plant phenological stage and grazing progress. Several correlations between insect counts and volatile compounds were found. Although their correlation coefficients were low, some of them were confirmed when tested by Spearman rank correlation, and could be logically explained. This method of grassland odorscape deserves to be developed because it can provide information on many aspects of grassland function and on the stresses that grassland plants undergo.

## Introduction

Biotopes such as corn fields, forests or grasslands are characterized by bouquets of fragrant volatile organic compounds (VOCs) that form their chemical signature, called “chemical landscape”, “odorant landscape” or “odorscape” by certain authors [1]. These compounds are emitted by plants to communicate with other plants, to defend themselves from attacks by herbivores or pathogens, and to attract pollinating insects ([2]; [3]).

Most of the studies conducted up to now have focused on the interaction between a given plant and a given insect, isolated in closed chambers to facilitate trapping and identification of the signal molecules. Recently, in a study by Leppik and Frerot [4] solid phase micro extraction (SPME) fibers were exposed within the canopy in a maize field, and the VOCs analyzed by gas chromatography mass spectrometry (GC-MS). The authors characterized the chemical landscape of the maize crop and showed that recognition of the plant by fertilized female moths was made possible by the detection of a fairly simple signature consisting of a mixture of VOCs.

Compared with a maize field, the chemical landscape of a semi natural grassland should be more complicated and difficult to capture. In Western Europe, semi natural grasslands are among those ecosystems richest in plant species and so potentially among the richest in VOCs. Extensive grazing has proved to be the most appropriate management option for favoring this plant diversity ([5], [6]). Decreasing grazing intensity favor not only plant but also arthropod diversity, because tall vegetation provides food resources in the form of nectar and pollen from flowers or live plant biomass and also shelter [7]. Hence grasslands form a reservoir for a variety of plants and a variety of associated arthropods among which the endangered wild bee species.

In this study, we set out to explore pasture odorscape as an innovative tool to assess and enhance the possible ecological roles and environmental services it offers. For example, identifying some indicators of wild bee populations among grassland VOCs could help to understand how the protective role of grasslands toward pollinating insects could be enhanced. Such work would certainly present a high level of technical difficulty. Given the great number of plant species that may occur in semi natural grassland compared with a maize field, and each plant species being low numbers, a given compound specific to a given plant will be secreted at a very low level. Moreover, in a maize field, the height of plants favors the existence of a protected microclimate that may concentrate its headspace, whereas grasslands are exposed to the wind, which even if slight, disperses the VOCs as soon as they are emitted.

We sought to test the followings hypotheses: (i) the same open air SPME-GC-MS method as used on maize field can be used to obtain the odorscape of a semi natural grassland, (ii) the chemical landscape of a botanically diverse semi natural pasture managed at a low stocking rate is more complex than that of a semi natural pasture with a moderate botanical diversity grazed at a higher stocking rate, (iii) changes in VOC profiles occur in a grassland odorscape when cows graze on it, owing to emission by plants of particular VOCs as a defense reaction, and finally (iv), grassland VOC profiles and the diversity of visiting wild bees are linked. Accordingly, VOC measurements were made in two mountain semi natural grasslands displaying contrasting abundances and diversities of flowering plants, at the beginning and just after an 8-day period of grazing.

## Material and Methods

### Experimental scheme

The experiment was conducted during July 2014 at the Inra farm of Marcenat (UEMA, UE1296) located in an upland area of central France on volcanic soils (45°18’N, 2°50’E; altitude 1070–1190m). The region is characterized by a low annual mean temperature of 7.4°C (2002–2013), and a high mean annual precipitation of 1167mm.yr^-1^, regularly distributed over the year. Mean temperature and precipitation in July are respectively 15.4°C and 108 mm with a global radiation of 2133 joules.cm-^2^ and a mean wind speed of 2.7m.s^-1^ (2002–2013). Two plots were used and chosen for their contrasting floristic diversity and management: "*Montagne*", a large 8.98 ha pasture that had received no fertilizer in the last 20 years and had always been grazed at a low stocking rate, resulting in a high botanical diversity, and "*La Prade*", a 1 ha pasture, flat and wet in places, well fertilized (30 UN.ha^-1^.yr^-1^ and 40 m^3^.ha^-1^.yr^-1^ of slurry) and grazed at a higher stocking rate, inducing moderate botanical diversity. The centers of the two pastures were 1.3 km apart and the highest point of *Montagne* and *La Prade* was respectively 1180 m and 1150 m. Measurements were made in July in two periods of 3 days corresponding to the flowering of the most abundant dicotyledons in botanically diverse grasslands. The first period P1 (1, 3 and 4 July) was when cows were grazing, and matched the second grazing rotation on *Montagne* and the third rotation on *La Prade*. The second period P2 (16, 17 and 18 July) was when cows had been turned out of the pastures (*Montagne and La Prade*: 10 July). On each pasture, three 10 × 12 m areas, representative of the different plant communities of the pastures in *Montagne* and *La Prade*, were excluded from grazing. On *Montagne*, two exclosures were set up on the slope (Exclosures 1 and 3) and one on the flat and more fertile area of the plot (Exclosure 2). On *La Prade*, one of the three exclosures was established in a wet place in the plot (Exclosure 1). Exclosures were removed just after the third day of measurement in each period. Wind speed, temperature, precipitation and humidity were recorded during P1 and P2 at the Inra meteorological station located on the farm. Botanical analysis, sward structure measurements, VOC trapping and insect counts were performed in triplicate in each of the six exclosures, in both periods unless otherwise stated.

### Botanical composition and vegetation structure

Botanical analyses were performed by a botanist (see Acknowledgments) at the end of P1, after insect and VOCs collection. All the species were determined in three 1 m^2^ quadrats distributed along the diagonal of each exclosure, and their abundances were scored on a 0−100 scale. Species covering less than 1% within a quadrat and species present in the exclosure but not in the quadrat were also identified. Abundance was calculated as the average abundance of each species identified in each quadrat. The Shannon index was used as a measure of plant species evenness at quadrat scale. Vegetation structure was measured at the end of the two periods at 50 locations over each exclosure. Every five steps, we recorded sward surface height, vegetation stage (leaf, stem or ear) and functional group (grass, legume or forb) at the first contact of a stick with the undisturbed sward surface. Flowering intensity was estimated based on the relative abundance of dicotyledonous (forb and legume) flowering stems. In addition, to globally depict the vegetation of each plot, we added height recordings using an electronic plate meter with a plastic plate (30 cm × 30 cm and 4.5 kg.m^-1^; FITC model/Arvalis), taking 60 measurements per hectare just before and after grazing, along transects distributed over the whole pasture. The standard deviation of sward herbage height was used as a vegetation heterogeneity indicator.

### Insect collection

Insects were trapped in each of the three exclosures each day of the two periods using pan traps [8]. The traps consisted of a set of three plastic cups, one white, one yellow and one blue, filled with water and a few drops of odorless detergent. The cups were attached facing south, at mean vegetation height (*Montagne*: 43 and 21 cm in P1 and P2 respectively; *La Prade*: 24 and 16 cm), on three stakes driven into the ground 5 m apart. At the end of the day, trapped insects were recovered by filtering through gauze and transferred to a labeled bottle containing 50% ethanol. Flies were discarded, wild bees were sorted, identified and counted, and the remaining hymenoptera were counted as “others”. As the traps failed to catch bumblebees because their weight and strength enabled them to escape, these were caught by nets for 15 minutes twice a day: between 11:00 and 12:00 and between 14:00 and 15:00 in each exclosure, each day of the two periods like the other measurements. They were also transferred to a labeled bottle containing 50% ethanol until identified.

### VOC analysis

#### Fiber conditioning and calibration

Twenty-four SPME fibers (DVB/CARBOXEN/PDMS 50/30 μm, Supelco^TM^, Bellefonte, PA) were cleaned by heating in a gas chromatograph injector at 250°C for five minutes with helium as the carrier flow. Cleaning efficiency had been previously checked in similar conditions: blank analyses confirmed that there was no carryover from the previous analysis. Fibers were controlled by GC-MS analysis after incubation in a headspace vial containing a mixture of the standard compounds ocimene, limonene, methyl salicylate caryophyllene and farnesene (Sigma-Aldrich, Saint-Quentin Fallavier, France). The variability expressed as the ratio between the mean and the standard deviation of compounds’ areas were 41, 13, 9, 22 and 30% respectively. Fibers were then wrapped in aluminum foil and stored in individual screw-capped Pyrex glass tubes until use.

#### Collection

The fibers were fixed on a metal stake in the middle of the exclosure, at the maximum canopy height (*Montagne*: 43 and 35 cm in P1 and P2 respectively; *La Prade*: 45 and 15 cm). They were positioned in the same place at P1 and P2. Each day of measurement, the 6 fibers were installed between 08:30 and 10:00 and removed in the same order between 16:30 and 18:00. Duplicate measurements were performed on the second day in P1, in order to measure individual variability. The second fibers were located within 4 cm of the first ones in each of the six exclosures. Fibers with trapped VOCs were wrapped again in aluminum foil and stored in individual screw-capped Pyrex glass tubes for transport to the laboratory in Versailles and analysis as described elsewhere [4]. Eighteen of the fibers that had been used in P1 were re-used in P2 after reconditioning.

#### Analysis

The SPME fibers were analyzed by gas chromatography coupled to mass spectrometry (GC/ MS) using a Bruker Scion 436-GC linked to a Bruker Scion SQ detector. The fused silica capillary column (30m×0.32mm i.d.) was coated with Rxi®-5SilMS (0.25 μm film thickness, Restek), the column temperature was programmed from 50 to 300°C at 8°C/min and carrier gas was helium N60 at constant flow of 2ml.min^-1^. Each SPME fiber was manually inserted in the injector and the VOCs desorbed in splitless mode, 5 min at 250°C. Mass spectra were recorded in the electron impact mode at 70ēV. Kovats’ retention indexes (RI) were computed using n-alkanes from C_10_−C_24_, eluted under the same conditions as the samples. Compounds were identified according to their retention index, to their molecular weight and to their mass spectra compared with the laboratory and with NIST 2011 library. VOC peak area information was extracted from the raw GC-MS data and transformed into relative amount by dividing the overall weight of the compound by the sum of the detected compounds from the same analysis and expressed as a percentage. A data matrix with relative amounts of all compounds for each individual was computed and aligned using GCAligner 1.0 [9]. The accuracy of the alignment was checked with original chromatograms.

### Data analysis

Statistical analyses were performed using Statistica Software (Statsoft, Maisons-Alfort, France). The dataset comprised VOCs and insect counts as variables and 36 observations: 2 pastures × 3 exclosures × 3 days × 2 periods. The duplicates performed on the second day of the first period constituted six additional observations. Measurements made on the second day allowed us to calculate a ratio between the mean of the duplicate and its standard deviation for each peak in each of the six exclosures. For each peak, the variability was expressed as the mean of the ratios from the six exclosures. The effects of the day of measurement and of the exclosure were tested separately by performing univariate ANOVAs. A two-way ANOVA was performed to test the effect of the grassland (G), the period (Per) and their interaction (G × Per) according to the model

$$X_{ij}=\;\mu +{\text{G}}_{i}+{\text{Per}}_{j}+{\left(\text{G}\times \text{Per}\right)}_{ij}+e_{n},$$

where *i* = *j* = 2 and *n* = 9

Post hoc mean comparison was performed using Fisher Least Square Difference (LSD) analysis. The effect of the grass and the effect of the period were checked using non-parametric, one-way ANOVAs of Kruskal-Wallis. A principal component analysis was performed using the variables for which the grassland and the period had the most significant effects. Correlations between VOCs and insects were tested using parametric statistical analysis and the highlighted relationships were checked using non-parametric Spearman rank correlations.

## Results

During P1, temperature was close to the average for July (15−17°C with a maximum at 25°C) but quite rainy with humidity and precipitation in the evening and night especially on the first and third days of measurement **(Table 1)**. Global radiation was high during the second day of measurement. In P2, weather was hot (18−22°C and a maximum at 29°C) with no precipitation. Global radiation was high throughout the period and humidity was low.

**Table 1:** Meteorological conditions during the two measurement periods.

	Day	MT	MaxT	MinT	P	W	GR	MH
**P1**	1	14.6	20.7	7.3	35	3.9	2166	103
	3	17.0	25.0	9.0	4	2.7	2592	89
	4	17.0	20.0	14.0	14	3.2	600	103
**P2**	16	17.8	26.0	9.1	0	2.8	2836	96
	17	21.4	27.6	13.5	0	3.6	3080	87
	18	21.7	29.0	14.7	0	5.2	2743	81

MT: mean daily temperature (°C), MaxT: maximum daily temperature (°C), MinT: minimum daily temperature (°C), P: daily precipitation (mm), W: mean wind speed (m.s^-1^), GR: global radiation (J.cm^-2^), MH: maximum humidity (%).

### Botanical composition

A total of 83 species were identified in the three exclosures on *Montagne* and 65 on *La Prade*, including respectively 63 and 43 dicotyledonous species. The quadrats on *Montagne* had a higher average number of species per square meter (30 *vs*. 19) and higher average Shannon indices (4.251 *vs*. 3.673) than those on *La Prade*. Forbs were much more abundant on *Montagne* than on *La Prade* (average 51% *vs*. 24%) while legumes, especially *Trifolium repens*, were more often found on *La Prade* than on *Montagne* (average 19% *vs*. 8%). Exclosure 1 and even more so Exclosure 3, located on the slope of *Montagne*, had higher species richness, forb richness and abundance and Shannon indices than Exclosure 2 located on the flat part (Table 2). In those two exclosures, plants were representative of oligotrophic species due to the low soil nutrient status of the slope. On *La Prade*, plants representative of hydrophilic species were present in Exclosure 1.

**Table 2:** Botanical diversity and species recorded on the two plots.

*Montagne*	Exclosure 1	Exclosure 2	Exclosure 3
Species per m^2^	32.3	22.0	36.0
Shannon index	4.341	3.620	4.791
Grasses %	24.1	68.4	31.7
Legumes %	7.7	8.7	7.5
Forbs %	68.2	22.9	60.8
Dominant species	*Thymus pulegioides s*.*l*. *(27%)*	*Festuca rubra rubra (25%)*	*Agrostis capillaris (14%)*
(Abundance ≥4%)	*Agrostis capillaris (6%)*	*Agrostis capillaris (19%)*	*Stachys officinalis (6%)*
	*Hieracium pilosella (5%)*	*Dactylis glomerata (14%)*	*Helianthemum nummularium (5%)*
	*Festuca rubra rubra (4%)*	*Thymus pulegioides s*.*l*. *(6%)*	*Chamaespartium sagittale (5%)*
	*Ranunculus bulbosus (4%)*	*Trifolium repens (4%)*	*Hypocheris radicata (5%)*
	*Galium verum (4%)*		*Meum athamanticum (4%)*
	*Hypocheris radicata (4%)*		*Achillea millefolium (4%)*
			*Leucanthemum vulgare (4%)*
			*Anthoxantum odoratum (4%)*
			*Hieracium pilosella (4%)*
***La Prade***	**Exclosure 1**	**Exclosure 2**	**Exclosure 3**
Species per m^2^	20.7	19.0	18.0
Shannon index	3.456	3.746	3.816
Grasses %	50.8	60.0	60.0
Legumes %	28.2	15.1	15.1
Forbs %	21.0	24.9	24.9
Dominant species	*Trifolium repens (28%)*	*Agrostis capillaris (14%)*	*Festuca rubra rubra (15%)*
(Abundance ≥ 4%)	*Juncus accutiflonis (15%)*	*Festuca rubra rubra (14%)*	*Trifolium repens (15%)*
	*Holcus lanatus (13%)*	*Trifolium repens (14%)*	*Plantago lanceolata (12%)*
	*Cynesorus christatus (6%)*	*Plantago lanceolata (11%)*	*Agrostis capillaris (9%)*
	*Ranunculus acris (6%)*	*Holcus lanatus (10%)*	*Dactylis glomerata (9%)*
	*Carex ovalis (5%)*	*Ranunculus acris (7%)*	*Lolium perenne (5%)*
	*Festuca rubra rubra (5%)*	*Taraxacum officinale (4%)*	*Ranunculus acris (5%)*
	*Ranunculus repens (5%)*	*Dactylis glomerata (4%)*	*Taraxacum officinale (5%)*
	*Polygonum bistorta (4%)*	*Lolium perenne (4%)*	*Cynesorus christatus (4%)*
			*Trisetum flavecens (4%)*

Grasses included *Carex sp*. and *Juncus sp*. Percentage represented the means of abundances recorded in the quadrats. Only species representing more than 4% of the abundance are presented.

### Vegetation structure

**Fig 1:**
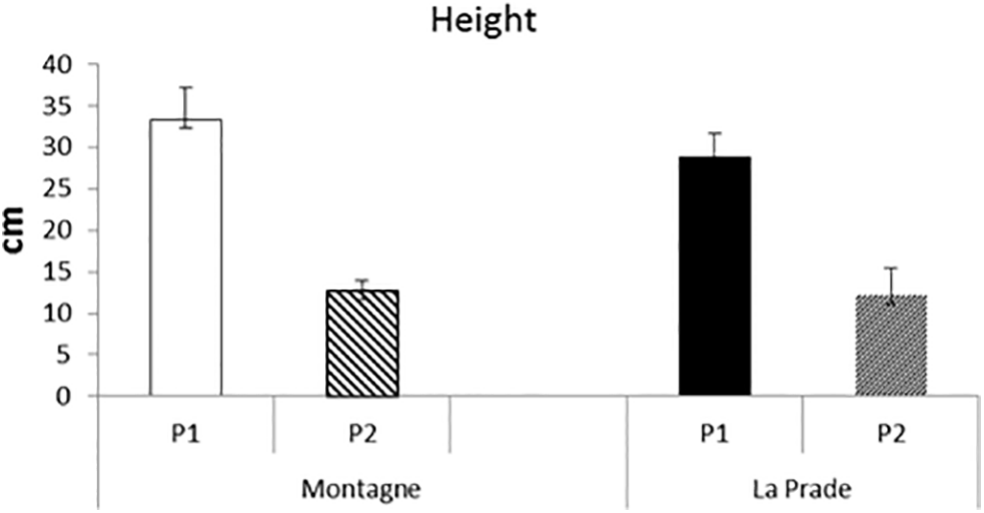
Sward surface height according to period and grassland.

**Fig 2:**
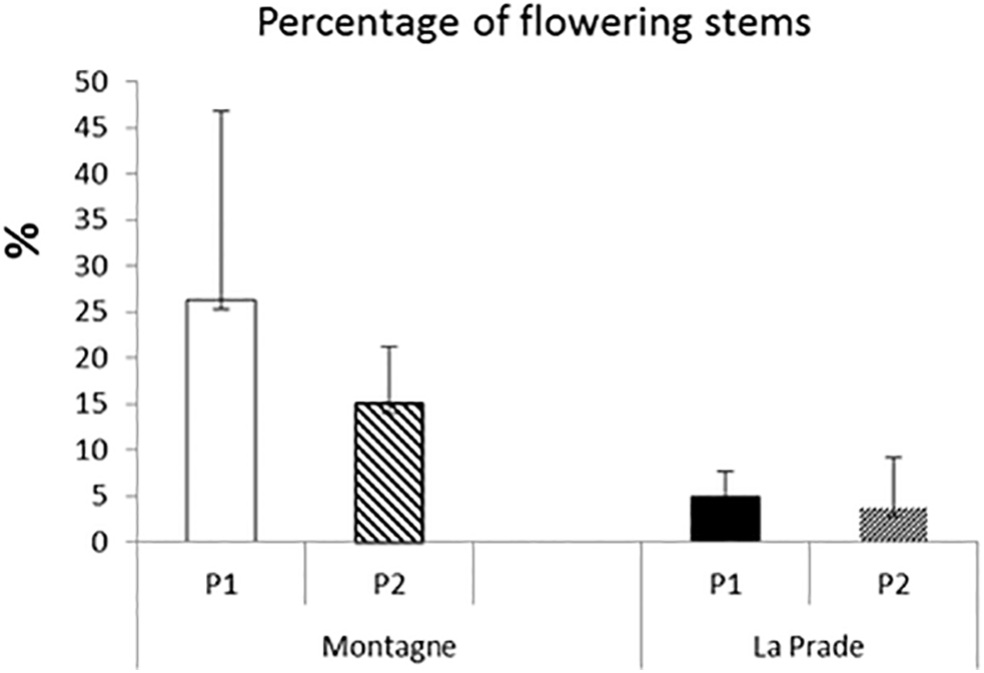
Percentage of flowering stems by period and grassland.

Sward surface heights within exclosures before and after grazing were similar between the two plots (**Fig 1**). Flowering intensity assumed by the percentage of dicotyledonous flowering stems was much greater on *Montagne* not only before but also after grazing compared with *La Prade* (**Fig 2**). Global herbage height before grazing was similar between *La Prade* and *Montagne* with a higher standard deviation (s.d.) on *Montagne*: 101 mm (s.d. 37.5 mm) *vs*. 102 (41.5). Decrease in height was greater on *La Prade* than on *Montagne* and the herbage cover was also less heterogeneous after grazing: 69 mm (s.d. 33.5 mm) *vs*. 95 (39.9).

### Volatile organic compounds

In total, 67 VOC were detected over the six exclosures and the two periods **(Table 3)**: 10 hydrocarbons, 8 alcohols, 10 aldehydes, 8 ketones, 7 acids, esters, lactones or anhydrides, and 24 unidentified compounds (UI). Among these were 13 benzene compounds and 11 terpenoids. The total number of compounds in the exclosures ranged from 63 to 66 on *La Prade* and from 60 to 67 on *Montagne*. These compounds were recovered on both plots, except for the UI-21 which was found only on *Montagne*. The total number of compounds was similar in P1 and in P2 (*Montagne*: 67, *La Prade*: 66). Lastly, four major compounds together represented 40% of the total peak area: UI-7, propan-2-one, 1-ethoxy-2-propanol and butyrolactone. Duplicate measurements performed on the 2^nd^ day gave a mean variability of 50% and a median value at 47% (data not shown). Fifty percent of the peaks had variability between 39 and 60%. The lowest variability value was 20% for Δ-caryophyllene and the highest 98% for UI-3 and butyrolactone. Despite this high variability, one-way ANOVAs highlighted 11 peaks significantly affected by day of measurement (p<0.001) and two by exclosure (p< 0.0001). The two peaks influenced by exclosure were the UI-14 and UI-21. These two compounds were found exclusively (UI-21) or in much higher proportions (UI-14) in Exclosure 3, on *Montagne*. The two-way ANOVA showed that six compounds were significantly more abundant on *Montagne* than on *La Prade*: terpinene, UI-3 and the UI-14 (p<0.01) propan-2-one, UI-21 and 2-phenylethyl acetate (p<0.05) (**Table 3**). Only benzoic acid was significantly more abundant on *La Prade* than on *Montagne* (p<0.05). A significant effect of the period was observed for 31 VOCs (**Table 3**). Among them, 19 compounds had a higher relative abundance in P1 than in P2: they included in particular the terpenes, 1-ethoxy-2-propanol, Δ-hexenol, Δ-hexenyl acetate, and 2-phenylethyl acetate. A smaller number of compounds (12) were more abundant in P2 than P1, among which most ketones, the aldehydes and 1-butanol. These effects were confirmed by the non-parametric Kruskal-Wallis one way ANOVAs performed separately on grassland and on period (not shown). Decanal was the only compound for which an interaction between the grassland and the period was observed: it tended to increase on *La Prade* and to decrease on *Montagne* between the two periods.

**Table 3:** Mean GC-MS peak areas of the VOCs adsorbed on carboxen-PDMS-DVB SPME fibers exposed in the two grasslands.

RI	Name	IQ	P1		P2				Effect	
			M	LP	M	LP	*SE*	P	G	P × G.
	***Hydrocarbons***									
869	Xylene	b	0.30	0.29	1.32	0.10	*0*.*60*			
1085	Δ-Ethylstyrene	c	2.47^a^	2.79 ^a^	1.43 ^b^	2.06 ^a^ ^,^ ^b^	*0*.*32*	**		
1116	*m*-Divinylbenzene	c	0.84	0.99	0.48	0.77	*0*.*18*			
932	α-Pinene	a	0.35	0.45	0.20	0.32	*0*.*12*			
1030	Limonene	b	0.20^a^	0.21 ^a^	0.09 ^b^	0.11^a^ ^,^ ^b^	*0*.*04*	**		
1058	Terpinene	b	0.19^b^	0.02 ^a^	0.14^a^ ^,^ ^b^	0.01^a^	*0*.*05*		**	
1411	Longifolene	b	0.11^b^	0.13^b^	0.04^a^	0.05^a^	*0*.*02*	***		
1421	Δ-Caryophyllene	b	0.29^b^	0.20^a^	0.14^a^	0.13^a^	*0*.*03*	**		
1481	Δ-Curcumene	b	0.11	0.12	0.08	0.09	*0*.*02*			
1508	Δ-Bisabolene	b	0.12^b^	0.11^b^	0.03^a^	0.02^a^	*0*.*03*	***		
	***Alcohols***									
640	1-Butanol	b	2.60^a^	2.53^a^	6.62^b^	5.94^b^	*0*.*47*	***		
741	2-Propanol, 1-ethoxy-	c	13.47^a^ ^,^ ^b^	15.62^b^	7.55^a^	8.61^a^	*2*.*22*	**		
850	Δ-Hexenol	a	0.49 ^a^	0.49 ^a^	0.32 ^a^ ^,^ ^b^	0.23 ^b^	*0*.*07*	**		
935	2-Propanol, 1-butoxy-	c	0.19	0.20	0.22	0.18	*0*.*04*			
1027	Ethylhexanol	b	3.04	4.71	2.74	2.73	*1*.*00*			
1032	Benzyl alcohol	b	2.25	1.84	3.93	3.06	*1*.*18*			
1244	2-Propanol, 1-phenoxy	c	0.44	1.33	0.31	0.38	*0*.*37*			
1290	Thymol	b	0.15^b^	0.10^a^ ^,^ ^b^	0.05^a^	0.05^a^	*0*.*02*	**		
	***Aldehydes***									
700	Pentanal	b	0.43 ^a^	0.48 ^a^	0.91 ^a^ ^,^ ^b^	1.29 ^b^	*0*.*17*	**		
780	3-Methyl-2-butenal	b	0.59	0.49	1.20	1.13	*0*.*46*			
800	Hexanal	b	2.81 ^a^	2.95 ^a^ ^,^ ^b^	4.27 ^b^	3.50 ^a^ ^,^ ^b^	*0*.*49*	*		
900	Heptanal	b	1.46 ^a^	1.56 ^a^ ^,^ ^b^	2.02 ^a^ ^,^ ^b^	2.15 ^b^	*0*.*23*	*		
958	Benzaldehyde	b	2.44	2.36	3.18	2.79	*0*.*32*			
1000	Octanal	b	2.33	2.29	1.95	3.31	*0*.*40*			
1104	Nonanal	b	3.76	3.74	4.46	4.81	*0*.*53*			
1180	4-Ethylbenzaldehyde	b	0.96	0.86	0.56	0.75	*0*.*13*			
1206	Decanal	b	3.08 ^a^ ^,^ ^b^	2.47 ^a^ ^,^ ^b^	1.85 ^a^	3.39 ^b^	*0*.*45*			*
1409	Dodecanal	b	0.20	0.19	0.13	0.17	*0*.*02*			
	***Ketones***									
626	propan-2-one	c	9.54^a^ ^,^ ^b^	5.20^a^	16.05^c^	11.67^b^ ^,^ ^c^	*1*.*96*	**	*	
883	3-Heptanone	b	0.14 ^a^	0.17 ^a^	1.18^b^	1.73^b^	*0*.*33*	***		
1064	Acetophenone	b	0.83	0.74	0.63	0.70	*0*.*11*			
1157	Sabinaketone	c	0.86	1.01	0.69	0.78	*0*.*13*			
1261	3,4-Dimethyl acetophenone	c	0.81	0.84	0.60	0.73	*0*.*09*			
1428	Δ-Diacetylbenzene	c	0.46	0.46	0.41	0.66	*0*.*08*			
1447	Δ-geranyl acetone	b	0.71^b^	0.60^a^ ^,^ ^b^	0.31^a^	0.50 ^a^ ^,^ ^b^	*0*.*11*	*		
1841	Hexahydrofarnesyl acetone	b	0.08 ^a^	0.08 ^a^	0.21 ^a^ ^,^ ^b^	0.35 ^b^	*0*.*06*	**		
	***Acids and derivatives***									
(632)	Acetic formic anhydride	c	3.83 ^a^ ^,^ ^b^	2.22 ^a^	4.32 ^a^ ^,^ ^b^	5.25 ^b^	*0*.*82*	*		
908	Butyrolactone	b	6.03	5.18	4.62	5.97	*3*.*02*			
1010	Δ-Hexenyl acetate	b	0.07^a^ ^,^ ^b^	0.09^b^	0.03^a^	0.04^a^ ^,^ ^b^	*0*.*02*	*		
1170	Benzoic acid	b	0.33^a^ ^,^ ^b^	0.39^a^	0.25^b^	0.38^a^	*0*.*04*		*	
1192	Methyl salicylate	a	1.49	2.67	0.82	1.25	*0*.*76*			
1253	2-Phenylethyl acetate	b	0.12^c^	0.09^b^	0.04 ^a^	0.02 ^a^	*0*.*01*	***	*	
1765	Benzyl benzoate	b	0.03	0.03	0.02	0.03	*0*.*01*			
	***Unidentified***									
722	UI-1		0.18	0.25	0.33	0.11	*0*.*08*			
759	UI-2		1.81	1.44	0.75	1.43	*0*.*51*			
790	UI-3		0.20 ^a^	0.11 ^a^	0.43 ^b^	0.22 ^a^	*0*.*04*	**	**	
836	UI-4		0.06 ^a^	0.07 ^a^	0.22 ^b^	0.18 ^a^ ^,^ ^b^	*0*.*05*	**		
860	UI-5		1.09	0.98	0.48	0.49	*0*.*25*	*		
864	UI-6		0.09	0.18	0.06	0.09	*0*.*04*			
890	UI-7		15.48	18.01	14.65	12.23	*2*.*59*			
992	UI-8		0.19^b^	0.20^b^	0.11^a^	0.09^a^	*0*.*03*	**		
1016	UI-9		0.07 ^a^	0.09 ^a^	0.15 ^a^ ^,^ ^b^	0.19^b^	*0*.*03*	**		
1050	UI-10		0.03^a^	0.03^a^	0.07^a^ ^,^ ^b^	0.09^b^	*0*.*01*	**		
1147	UI-11		0.57	0.41 ^b^	0.35 ^a^	0.28	*0*.*08*			
1163	UI-12		2.24	2.52	1.75	1.76	*0*.*30*	*		
1182	UI-13		1.74 ^b^	1.69 ^b^	0.91 ^a^	1.18 ^a^	*0*.*16*	***		
1187	UI-14		0.21 ^b^	0.08 ^a^	0.09 ^a^	0.03 ^a^	*0*.*03*	*	**	
1201	UI-15		1.11 ^b^	0.98 ^b^	0.50 ^a^	0.64 ^a^	*0*.*11*	***		
1230	UI-16		0.57^b^	0.53^a^ ^,^ ^b^	0.30^a^	0.3^a^	*0*.*08*	**		
1270	UI-17		0.71 ^b^	0.50 ^a^ ^,^ ^b^	0.26 ^a^	0.19 ^a^	*0*.*14*	*		
1276	UI-18		0.23	0.20	0.16	0.18	*0*.*03*			
1282	UI-19		1.30	1.31	0.80	1.13	*0*.*18*			
1303	UI-20		0.33	0.39	0.25	0.34	*0*.*05*			
1325	UI-21		0.14^b^	0.00^a^	0.02^a^	0.00^a^	*0*.*04*		*	
1337	UI-22		0.51	0.56	0.43	0.48	*0*.*11*			
1360	UI-23		0.10	0.10	0.07	0.10	*0*.*02*			
1612	UI-24		0.02	0.09	0.49	0.11	*0*.*23*			

Mean areas are indicated in percentage of the total area. Fibers were exposed in *Montagne* (M) and *La Prade* (LP) grasslands at the beginning (P1) and after (P2) cows grazing. Significance of the effects of the grassland (G), the period (P) and their interaction (G × P)

***p<0.001

**p<0.01

*p<0.05.

IQ: identification quality

a MS and RI in accordance with published databases and with the laboratory database

b: MS and LRI in accordance with published databases and c: MS in accordance with published databases, RI not available. Groups in the same row sharing the same superscript do not differ significantly according to Fisher Least Square Difference (LSD) analysis (*p* < 0.05).

**Fig 3:**
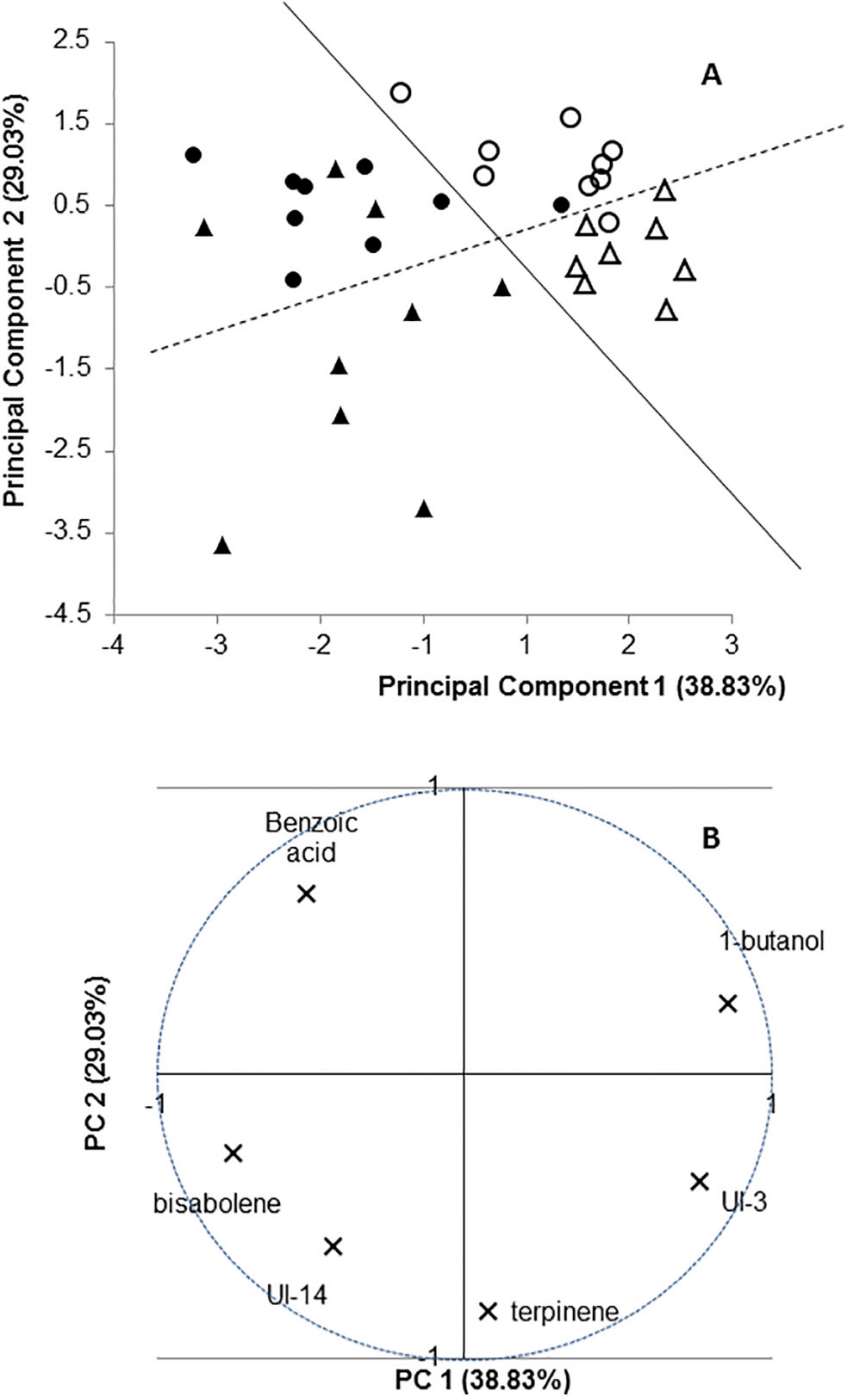
Representation of *Montagne* and *La Prade* grasslands at both periods on the 1 × 2 plane of the PCA. A: Observations (2 grassland× 3 exclosures × 3 repetitions× 2 periods) and B: Variables included in the PCA. The first Component separated period 1 (filled symbols) from period 2 (empty symbols), while the second Component separated *Montagne* (triangles) from *La Prade* (circles).

The PCA performed with the peaks most significantly influenced by the grassland (terpinene, UI-14 and benzoic acid), the period (1-butanol and Δ-bisabolene) or both (UI-3), is presented in **Fig 3**. Together, the two first components explain 68% of the variance. Principal Component 1 (PC1) separates P1 to the left from P2 to the right. This is mainly due to β-bisabolene and UI-14 which have higher relative abundances in P1. The two plots tend to separate along the second Principal Component (PC2), with *La Prade* above and *Montagne* below the dotted line except for four individuals: three from *Montagne* (P1 − Exclosure 1 − day 2, P1 –Exclosure 2 − day 2, P1 − Exclosure 3 − day 1) and one for *La Prade* (P2 − Exclosure 2 − day 2).

### Insects

Bumblebee numbers were significantly higher on *Montagne* than on *La Prade* **(** *p*<0.05, **Table 4)**, a difference that was also significant for the number of bumblebee species (*p*<0.01), and for the number of the wild bee species caught in the pan trap (*p*<0.05). On *Montagne*, the wild bee group was representative of a diversified thermophilic mountain pasture, while on *La Prade* the group reflected ordinary semi-natural grassland. No difference on the number of other hymenoptera was found.

**Table 4:** Insects trapped in the two plots.

	P1		P2			Effect		
	M	LP	M	LP	*SE*	Period	Plot	Per.× Plot
Number of bumblebees	1.44	0.78	2.44	0.00	*0*.*58*		*	
Number of bumblebee species	0.78	0.56	1.78	0.00	*0*.*36*		**	*
Number of wild bees	1.56	0.44	1.11	0.56	*0*.*41*			
Number of wild bee species	0.89	0.44	1.00	0.44	*0*.*25*		*	
Number of other hymenoptera	1.56	0.78	1.89	1.78	*0*.*53*			

Insects were trapped in the two periods (P1 and P2) with the net (bumblebees) or with the pan trap (wild bees and other hymenoptera) in *Montagne* (M) and *La Prade* (LP) plots. Data are the represented means of nine counts (3 days × 3 exclosures).

Significant correlations (*p*<0.05) between VOCs and insect counts, found using either the values or their rank are presented in **Table 5**. Whatever the method, there was a negative correlation between the number of wild bee species and limonene (R = -0.39) and between the number of “other hymenoptera” and α-pinene (R = -0.35), Δ-hexenyl acetate (R = -0.41) and limonene (R = -0.46).

**Table 5:** Correlations between VOCs and insects.

		Bumblebees	Bumblebee species	Other hymenoptera	Bees	Bee species
**Value correlation only**						
Benzaldehyde			0.37			
Butyrolactone				0.36		0.34
**Rank (Spearman) correlation only**						
Acetophenone					0.39	0.35
Δ-Curcumene				-0.33		
Limonene					-0.36	
UI-21			0.43			
Methyl salicylate				-0.34		
xylene		-0.34	-0.40			
UI-16					0.37	0.34
**Value and rank correlations**						
α-Pinene	value			-0.35		
	rank			-0.35		
*Δ*-Hexenyl acetate	value			-0.41		
	rank			-0.57		
Limonene	value			-0.46		-0.39
	rank			-0.53		-0.41

Other hymenoptera correspond to the hymenoptera remaining after removing flies and wild bees from the pan-trap. Coefficients (R) found significant (*p*<0.05) when tested using relative abundance and count values or using ranks according to Spearman.

## Discussion

The SPME-GC-MS method of Leppik and Frerot [4] provided grassland VOC profiles, supporting our starting hypothesis. As expected, compared with the VOC profiles of maize cultures [4], the VOC profiles of the grasslands contained a greater number of compounds, belonging to a wider range of chemical families. Five VOCs were common with those found in maize [4]: the two terpenes α-pinene and limonene, methyl salicylate and the green leaf volatiles *Δ*-hexenol and *Δ*-hexenyl acetate. These compounds are very common semiochemicals that are systematically observed in plant—herbivore interaction studies and known as herbivore induced plant volatiles (HIPVs) ([2], [10]). Despite the presence of a high percentage of terpene-rich plant like *Thymus pulegioides*, *Meum athamanticum* and *Achillea millefolium* recorded on *Montagne* [11], the number of terpenes was lower (12 *vs*. 16) than in the maize field odorscape [1], probably a result of individual plant dilution. The most abundant VOCs in our profiles, UI-7, propan-2-one, 1-ethoxy-2-propanol and butyrolactone were not observed in the maize field odorscape. Propan-2-one may have an abiotic origin as well as a microbial, plant or animal origin [12]. It is also known as a signal molecule toward insects [13]. The compounds originating from ruminants that could have been expected, such as volatile fatty acids from C_2_ to C_7_, sulfur compounds, phenolic compounds and indole derivatives ([14], [15]), were not found. Only benzyl alcohol and benzoic acid were recovered, and this compound could also have originated from plants. Apart from benzoic acid, no carboxylic acid was found among the VOC profiles. This was probably not due to a poor affinity between these compounds and the phases on the SPME fibers, as headspace extraction of volatile fatty acids on carboxen-PDMS-DVB has already been described [16]. Another hypothesis is that some of the diverse alcohols, ethers, aldehydes and esters that occurred in our profiles originate from volatile fatty acids although we could not find any report on such reactions occurring in open air.

The trapping conditions were probably less efficient than in maize crops, where the headspace may be protected against wind, retained and to some extent concentrated by the height of the plants. A relatively high variability was indeed observed between the duplicate measurements made a few centimeters away on the second day of P1. This variability certainly stemmed from many sources, e.g. the heterogeneous distribution of the plants producing VOC in the grassland and a sporadic delivery of these VOCs, depending on the interactions of the plants with their environment. Moreover, VOC concentration in the air depends greatly on physicochemical conditions, mainly temperature, more compounds becoming volatile as temperature increases and above all, more VOCs being emitted by plants. In addition, VOC in the open air may disappear due to chemical reactions, or simply in the air flows. Exposing the fibers all day long probably narrowed this variability, each profile representing an average of the chemical landscapes encountered at one point.

Although the two plots, chosen due to their contrasting management, had different botanical compositions, these differences did not induce broad variations in their VOC profiles. The number of compounds on *Montagne* was appreciably the same as on *La Prade*, despite the higher proportion of forbs and the higher level of botanical diversity, expressed by a higher Shannon index on *Montagne*. The great heterogeneity between exclosures in terms of botanical composition induced little changes in VOC profiles except for Exclosure 3 on *Montagne*, which was characterized by UI-14 and UI-21. Marked differences in VOC relative percentages were observed between P2 and P1, the total number and the nature of compounds remaining sensibly the same. The effects of temperature and humidity changes between P1 and P2 merge with those of plant maturation and cow grazing. Among the compounds that were more abundant in P1 when cows were grazing beside the exclosures, compounds such as longifolene and bisabolene probably reflected the higher percentage of dicotyledonous flowering plants in P1 compared with P2. Herbivore induced plant volatiles are emitted when plants are attacked by herbivorous insects ([2], [10]). In our experiment, the HIPVs Δ-hexenyl acetate and Δ-hexenol could reflect the presence of the grazing cows. Compounds that had higher relative abundance in P2 than in P1 were 1-butanol and 3-heptanone, which could have a plant as well as an animal origin. Finally, the two periods were well discriminated on the PC1× PC2 plane off the PCA (Fig 3), and there was a trend for the two plots. Benzoic acid characterized *La Prade* while terpinene characterized *Montagne*, together with UI-14 in P1 and UI-3 in P2.

Wild bee populations were markedly different in the two plots. Wild bees therefore reliably distinguished between the two plots whereas recording of the chemical odorscape barely differentiated them. This flower- visitor’s power of discernment has already been underlined by Hegland and Boeke [17], and Sjodin [6] on a plot scale. Interesting relationships are nevertheless highlighted between VOCs and wild bee populations. Although these relations clearly need to be confirmed, they provide clues to finding “semiochemicals” linking grasslands to the insects they host, such as benzaldehyde, positively correlated with the number of bumblebee species, already known as a semiochemical. Butyrolactone, which correlated with the number of pollinators and the number of bee species, has also been cited as a constituent of insect pheromones[18]). Lastly, negative correlations between limonene and the number of wild bee species observed in our study, could reflect the vegetative stage of the plants, limonene being largely emitted by leaves and stems rather than by flowers. Thus, this method provided Leppik and Frerot [4] a better understanding of the behavior of the corn borer in a maize field and brought new knowledge to the fight against this crop pest. In our case, VOC profiles could help determine which plant species are the most attractive or unattractive for wild bees and other pollinators and consequently which are those plants whose presence is important in maintaining flower-visiting insects.

## Conclusion

This study has shown that the SPME-GC-MS method performs well enough to collect VOCs from a grassland atmosphere and to obtain the grassland odorscape. This volatilome contains a variety of compounds and constitute a starting library of chemical landscapes for further studies. Future improvement of the method should permit to plainly differentiate the two experimental grasslands, which were very different in their botanical composition. In addition, a different experimental scheme should be applied to highlight changes in the VOC profiles attributable to grazing and differentiate them from those of the ambient temperature and plant ageing. Comparison between two grasslands implies measuring chemical landscapes with similar meteorological conditions.

Odorscape recording brings new knowledge and offers tools to assess and enhance grassland value in terms of ecological services. In this respect, the correlation between wild bees and certain VOCs in the profiles are encouraging. VOC profiles could give indicators of microbial activity in soil or pollinating attractiveness. In the future, relationships between grasslands odorscape and milk sensorial quality would also be an interesting track to explore.
